# Reducing disability via a family centered intervention for acutely ill persons with Alzheimer’s disease and related dementias: protocol of a cluster-randomized controlled trial (Fam-FFC study)

**DOI:** 10.1186/s13063-018-2875-1

**Published:** 2018-09-17

**Authors:** Marie Boltz, Ashley Kuzmik, Barbara Resnick, Rebecca Trotta, Jacqueline Mogle, Rhonda BeLue, Douglas Leslie, James E. Galvin

**Affiliations:** 1 The Pennsylvania State University, College of Nursing, 306 Nursing Sciences Building, University Park, PA 16802 USA; 2 University of Maryland School of Nursing, Baltimore, MD USA; 3 Hospital of the University of Pennsylvania, Philadelphia, PA USA; 4 St. Louis University, St. Louis, MO USA; 5 Florida Atlantic University, Boca Raton, FL USA

**Keywords:** Dementia, Hospitalization, Post-acute, Family engagement, Functional recovery

## Abstract

**Background:**

Hospitalized older persons with Alzheimer’s disease and related dementias are at greater risk for functional decline and increased care dependency after discharge due to a combination of intrinsic factors, environmental, policy, and care practices that restrict physical and cognitive activity, lack of family involvement and limited staff knowledge of dementia care. We have developed a theory-based intervention, Family centered Function-focused Care, that incorporates an educational empowerment model for family caregivers (FCGs) provided within a social-ecological framework to promote specialized care to patients with dementia during hospitalization and the 60-day post-acute period. Primary aims are to test the efficacy of the intervention in improving physical and cognitive recovery in hospitalized persons living with Alzheimer’s disease and related dementias (ADRD) and improving FCG preparedness and experiences.

**Method:**

We will implement Family centered Function-focused Care in a cluster-randomized trial of 438 patient/FCG dyads in six hospital units randomized within three hospitals. We hypothesize that patients who receive the intervention will demonstrate better physical function, less delirium occurrence and severity, neuropsychiatric symptoms, and depression compared to those in the control condition (Education-only). We also hypothesize that FCGs enrolled in Family centered Function-focused Care will experience increased preparedness for caregiving, and less strain, burden, and desire to institutionalize, as compared to FCGs the control group. We will also examine the costs and relative cost savings associated with the intervention and will evaluate the cultural appropriateness of Family centered Function-focused Care for families from diverse backgrounds.

**Discussion:**

Our theory-based intervention makes use of real-world applicable approaches in a novel and innovative way to change the paradigm of how we currently look at acute care and post-acute transitions in persons with ADRD.

**Trial registration:**

ClinicalTrials.gov, ID: NCT03046121. Registered on 8 February 2017.

**Electronic supplementary material:**

The online version of this article (10.1186/s13063-018-2875-1) contains supplementary material, which is available to authorized users.

## Background

Approximately one in nine people age 65 years and older has Alzheimer’s disease or a related dementia (ADRD), increasing to about one third of people by age 85 years [1]. Many of the estimated 5.5 million older adults with ADRD also suffer from one or more serious medical conditions, including coronary artery disease (26%), diabetes (23%), and chronic obstructive pulmonary disease (15%) [1, 2]. These co-existing medical conditions as well as other potentially high-risk, acute issues (e.g., falls, infections, medication side effects, etc.) contribute to the high prevalence of hospital stays in this group [1, 2]. As a result, persons with dementia are twice as likely to be hospitalized as persons without dementia and comprise one fourth of hospitalized older adults [1, 3, 4].Once hospitalized, individuals with ADRD are more likely to experience potentially preventable complications such as delirium, neuropsychiatric symptoms (NPS), pressure ulcers, falls, and nutritional deficiencies [5–8]. In addition, they often have clinically significant functional decline resulting in increased hospital costs (twice as much for persons with dementia) [9], morbidity, and earlier mortality [10–14]. During the post-acute period, persons with ADRD are more likely to experience protracted delirium with increased care needs and lower quality of life for both themselves [15] and their family caregiver (FCG) [16–18]. Further, they utilize more post-acute care [19] and are at increased risk for re-hospitalizations, transitions to long-term nursing home stays, and mortality than persons without ADRD [10, 14, 20–22]. Consequently, at 6 months post discharge, individuals with ADRD are 600 times more likely to be in a nursing home [22] compared to cognitively intact, community-dwelling older adults prior to hospitalization, and few return to pre-hospital functional status [20].

In hospital settings, acute illness superimposed upon baseline cognitive and functional vulnerabilities account for only some of the physical and cognitive declines associated with hospitalization of the person with ADRD [23, 24]. Other factors associated with negative outcomes include: (1) lack of dementia-sensitive care processes (i.e., inadequate assessment of patient and FCG needs and preferences) [25, 26]; (2) activity-restrictive policies and environments [27–30]; (3) staff under-prepared to care for persons with cognitive impairment [31, 32]; and (4) patient, staff, and FCG attitudes regarding physical activity in the acute setting [33–35].

Prior to hospitalization, approximately 75% of hospitalized patients with ADRD are living at home and receiving care from family members or friends [20, 36, 37]. Upon hospitalization, the goal of most families is to have their relative return home [15]. However, FCGs are limited in the amount of physical care they can provide in terms of lifting, moving, and providing incontinence care [17, 18]. The additive stress of the hospitalization, and the patient’s increased functional dependency, often co-existing with persistent delirium, compounds the strain of the FCG, prompting the decision to seek long-term nursing home care [18, 38]. It is particularly important, therefore, that patients with ADRD receive the type of care, beginning at admission and continuing through the post-acute transitional period, that optimizes cognitive and physical function and physical activity and helps prevent or decrease functional decline.We have developed a theory-based intervention that adapts Function Focused Care (FFC) to the hospitalized person with dementia. Function Focused Care is a philosophy of care in which caregivers (formal and/or informal) are taught to care for patients in a way that engages in them in performing functional tasks and physical activity at their highest levels [39]. Fam-FFC builds on this approach by incorporating the family and adapting FFC to the specialized needs of persons with ADRD. Specifically, Fam-FFC provides expert dementia care by formal caregivers (nursing staff) in the acute care setting through adaptations to the physical and care environment but, importantly, incorporates the additional component of the patient’s FCG. In this family centered care approach, nurses purposefully engage FCGs in the assessment, decision-making, discharge planning, care delivery, and evaluation, beginning at admission, continuing through the hospital stay and the 60-day post-acute period. Fam-FFC provides a unique opportunity for skill development, education and support in the care needs of care recipients and helps FCGs gain confidence in encouraging and facilitating optimal function and physical activity of the patient with ADRD.

In this paper we describe a National Institute of Aging (NIA)-funded research protocol for a cluster randomized clinical trial designed to test the efficacy of Fam-FFC. We describe: the intervention rationale, the theoretical framework that guides the study, the specific aims, and the methods.

### Intervention rationale

When engaged to do so, FCGs of persons with ADRD provide essential information on baseline cognitive and functional status necessary to guide care delivery and track treatment response [40, 41]. Although not typical practice in the acute care setting, discussions of patients’ needs and preferences between nurses and the patient’s close family members have been found to be useful in predicting and preventing excessive stress in persons with dementia. This type of person-centered care approach has the potential to prevent inappropriate use of antipsychotics and mitigate progressive physical or cognitive decline [41–43]. Furthermore, family members can play a positive role by motivating and encouraging patients during the hospitalization [33–35] and subsequently at home [44].

Although hospitalization may be expected to provide respite for FCGs, the experience is often associated with increased stress for these individuals [17, 45]. The patient’s functional loss, presence of delirium and neuropsychiatric symptoms increase FCG burden [45]. Additionally, the pre-existing, chronic strain borne by FCGs of persons with ADRD is compounded by anxiety about the comfort and safety of the patient during their hospital stay and the potential for increased care needs at discharge [45–48]. The combination of the patient’s care needs (activities of daily living (ADLs) dependency, depression, behavioral symptoms) and caregiver challenges (preparedness, burden, strain) are associated with the decision to seek long-term care placement [38, 49]. Interventions that improve the cognition, physical function, and emotional response of the patient can help improve FCG outcomes and mitigate the desire to institutionalize the care recipient.

The family member’s baseline knowledge and understanding of disease processes, optimal care approaches and prevention of adverse events are varied and may not be consistent with current evidence-based care approaches [50–53]. Particularly, a belief that restricting physical activity will prevent falls and a lack of understanding of the ramifications of inactivity and subsequent deconditioning and functional loss may cause families to restrict physical activity of the care recipient during the acute care stay [35]. Efforts to productively involve FCGs in function-promoting care requires a concerted effort to provide information, an appraisal of the FCG’s preferences for involvement, and a systematic process of engagement in goal setting and ongoing decision-making during acute and post-acute care. Pilot work demonstrated that Fam-FFC is feasible to implement for hospitalized persons with ADRD. We found that hospital nursing staff can integrate FFC into their practice and that FCGs can adapt this approach in their various role functions [54, 55].

### Aims

The aims of this trial are: aim 1: validate the efficacy of Fam-FFC on patient outcomes: physical function (ADLs/ performance and physical activity), delirium occurrence and severity, NPS, and mood; aim 2: evaluate the impact of Fam-FFC on FCG-centered outcomes (preparedness for caregiving, strain, burden, and desire to institutionalize); and aim 3: calculate the costs of Fam-FFC, evaluate relative health care costs (post-acute health care utilization) and total cost savings for Fam-FFC v. the control condition. We also evaluate the cultural appropriateness of Fam-FCC for diverse families in our sample.

### Theoretical basis of Fam-FFC

**Fig. 1:**
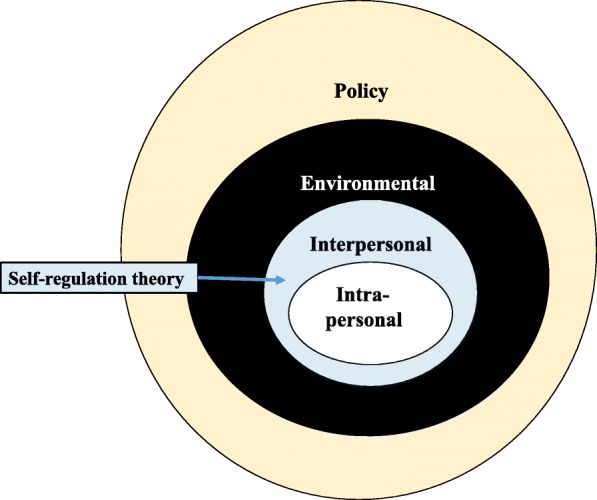
Social Ecological Model: factors influencing functional recovery in hospitalized persons with dementia

The Fam-FFC intervention was developed using a social ecological framework [56] that acknowledges the intra-personal (intrinsic), interpersonal (related to nursing staff, patient, family interaction), and environmental and policy/process factors that influence function in hospitalized persons with dementia (see Fig. 1). Social Cognitive Theory (SCT) is used at the interpersonal level to facilitate behavior change [57]. SCT suggests that the stronger the individuals’ self-efficacy and outcome expectations, the more likely it is that they will initiate and persist with a given activity. Self-efficacy expectations are the individuals’ beliefs in their capabilities to perform a course of action to attain a desired outcome; and outcome expectations are the beliefs that a certain consequence will be produced by personal action. Efficacy expectations are dynamic and enhanced by four mechanisms: (1) successful performance of the activity; (2) verbal persuasion; (3) seeing like individuals perform a similar activity; and (4) pleasant physiological and affective states (e.g., pain reduction) associated with an activity [58]. These four mechanisms are incorporated into Fam-FFC to motivate patients to be functionally and cognitively active; education and coaching are provided to both staff and FCGs to utilize these mechanisms. Fam-FFC acknowledges that nurses are ideally positioned to integrate FFC care within their daily interactions with patient and families, and to support the FCG role in FFC [54, 55]. Thus, the Family centered Function Focused Care nurse (Fam-FFC nurse) involves the staff nurse, along with the patient and FCG, in the care planning, delivery, and evaluation process of Fam-FFC interventions.

## Methods

### Design

The experimental design for this study is a longitudinal design with three follow-up assessments post discharge where study units are randomized by clustering to either treatment (Fam-FFC) or the control condition. The control condition (Fam- FFC Ed-only) consists of education of the nursing staff with no other intervention. This design allows a within- and between-group analysis to ascertain the effects of Fam-FFC. The experimental condition, Fam-FFC, is coordinated and implemented by a Family centered Function Focused Care Nurse (Fam-FFC Nurse), who works in the treatment units 35 h a week (on the unit 7 days a week) for 14 months to implement the four components of the intervention. In addition, the sites identify two members of the nursing staff from each unit (to cover both day and evening shifts) to be Fam-FFC champions who support the logistics and sustainability of the intervention.

### Settings

Six inpatient units of three hospitals (two units each), located in the Northeast USA and 340 hospitalized persons and their FCGs are included in the study. To improve the potential for translation into practice and to increase the generalizability of our findings hospitals represent three distinct types and bed size: a large academic medical center, a medium-sized teaching hospital, and a small community hospital. Sites were selected to ensure a large population for recruitment and to increase the diversity of the sample. The sites have committed to engagement of the nurse champion in the intervention as well as the evaluation of the cultural appropriateness of Fam-FFC.

### Eligibility criteria

Patient inclusion criteria includes age ≥ 65 years, speak English or Spanish, live in the community prior to admission to the hospital, screen positive for dementia on well-validated scales (Montreal Cognitive Assessment (MoCA) ≤ 25 [59–63] and AD8 ≥ 2 [64, 65]), have a diagnosis of very mild to moderate stage dementia as confirmed by a score of 0.5 to 2.0 on the Clinical Dementia Rating Scale (CDR) [66], and have a FCG as the designated study partner for the duration of the study. Patients are excluded from the study if they have mild cognitive impairment (CDR = 0.5 without functional or ADL impairments), severe dementia (CDR =3), any significant neurological condition associated with cognitive impairment other than dementia (e.g., brain tumor), a major acute psychiatric disorder, have no FCG to participate, are enrolled in a hospice, are admitted from a nursing home, or experience transfers to another unit for stays longer than 48 h.

FCGs age 18 years and above are eligible if they speak English or Spanish, are related to the patient by blood, marriage, adoption, or affinity as a significant other (defined by the legally authorized person as the primary person providing oversight and support on an ongoing basis), are able to recall at least two words of a three-word recall (to eliminate cognitive impairment), and participate, at a minimum, in the initial assessment and development of the plan of care.

For the exploratory aim of assessing the cultural appropriateness of the intervention, we recruit FCGs who self-identify as black, Latino, Asian and white, randomly selected from the Fam-FFC sample. Approximately 10% of families from each ethnic group represented in the study are approached for consent for participation in interviews. (Interviews continue until theoretical saturation is reached). Additionally, the six registered nurse (RN) champions are consented and interviewed after the study ends in their particular unit/setting to provide their perspective on the cultural appropriateness of Fam-FFC. Finally, at the conclusion of the intervention at each site, approximately six to eight RNs who have worked on the study units and represent both shifts are recruited to participate voluntarily in focus groups evaluating nurse-reported perceptions of the influence of management and organizational culture, the likelihood of continuing to implement the intervention, and satisfaction.

## Recruitment, enrollment, and randomization

### Recruitment and enrollment

Research staff work with identified setting staff to provide a list of potentially eligible patients (age ≥ 65 years, dementia, not in a hospice or admitted from a nursing home). The patient and legally authorized representative (LAR) are approached to discuss the study, and if granted, receive: (1) brief oral and written information about the study and (2) a copy of the consent form. The requirement to have a FCG involved and consented is explained to the patient/LAR. The patient and LAR may decide that another person, a FCG other than the LAR, is the best person to participate in the study. In that case, that FCG is provided information and, if agreeable, engaged in the consent process. Both patient and FCG consent are required. After information is provided, and the patient and FCG agree to participate, an evaluation of capacity to provide consent is conducted, using the Evaluation to Sign Consent (ESC) [67]. If capacity is determined the patient may provide their own consent. If decisional capacity is impaired, and the patient provides assent, the LAR completes the consent process. The LAR is encouraged to try and determine what the patient would do if competent and base their decision upon what they think is in the patient’s best interest. The FCG’s ability to sign consent is also evaluated using the ESC. Only FCGs answering all ESC questions correctly are enrolled. Both patient and FCG (who may or may not be the LAR) consent are required for the study process to proceed to screening.

Upon consent, the research evaluator checks FCG eligibility and then screens the patient for dementia using the Montreal Cognitive Assessment (MoCA) [59] and AD8 [64]. Patients who screen positive for dementia are then assessed for severity of dementia using the CDR [66]. A diagnosis of very mild to moderate stage dementia as confirmed by a score of 0.5 to 2.0 on the Clinical Dementia Rating Scale is required. Functional ability is assessed with the Pfeffer Functional Activities Questionnaire (FAQ) to discriminate mild cognitive impairment (MCI) from dementia [68].

The consent process of patient/FCG dyads occurs within 48 h of admission to the unit. Patients are consented in person. The FCGs are also consented in person unless they are unable to be present with the research staff within the 48-h timeframe, in which case they are consented over the telephone. At the beginning of the study, prior to patient/FCG enrollment, nurse champions are consented to participate in an interview conducted after the intervention on their respective units to evaluate the cultural appropriateness of the intervention. Hospital staff nurses are consented for post-intervention focus groups at the conclusion of the intervention. The focus groups explore their perceptions of the intervention, including its sustainability.

### Randomization plan

The level of engagement required for the intervention prevents random assignment at the patient level, as patients would be alerted to differential treatment within the same unit and there is the risk of treatment “contamination” between intervention and control participants. Patients cannot be randomly assigned to inpatient units as assignment is determined by hospital staff, based upon the services required for each patient, as well as other factors. Thus, after the hospitals are randomized to the time cohort, within each hospital the two inpatient units are randomly assigned so that one unit receives Fam-FCC and the other unit receives the Staff-Ed (control) intervention. The risk of contamination is minimized by the fact that the intervention and control hospital units are not adjacent to one another; and are separated by several floors in one hospital and in two of the hospitals, the units are in different buildings. Nursing care units have separate nursing staff and management, and nurses do not rotate between units. Further, the enrolled patients and FCGs do not share communal areas between the units, and the units do not share nursing staff. Random assignment is completed by the statistician using SAS proc. plan which specifically allows simultaneous randomization on multiple dimensions (time and units), as well as counterbalancing across units. Participants are blinded to treatment arm prior to consent. Evaluators are not informed of randomization results or provided with the details of the intervention.

## Description of the Fam-FFC intervention

The four components of Fam-FFC implemented by the Fam-FFC nurse, include: Component I – Environmental and Policy Assessments; Component II – Education and Training of Nursing Staff; Component III – Implementation of the FamPath Care Pathway, and Component IV –Ongoing Training and Motivation of Nursing Staff. The Environmental and Policy Assessment is designed to evaluate the unit’s readiness to implement Fam-FFC, including staff and family access to supplies, practices that support family engagement in care and decision-making, and general features that support comfort, safety, and function of older adults with cognitive impairment. Component II, education and training of the staff, focuses on the specialized needs of the person with dementia and their family members, how to provide and integrate FFC into all routine care, delirium prevention, detection, and management, and strategies to partner with FCGs to facilitate the care of the patient with dementia. Components I and II are implemented over a 2-month period, prior to enrolling patients and FCGs.

The Fam-FFC nurse initiates the FamPath care pathway, Component III, upon enrollment of the patient/FCG. Information and education are provided to the patient and FCG and the Fam-FFC nurse collaborates with the FCG /patient, with input from the interdisciplinary team to develop interdisciplinary bedside goals based on the underlying capability of the patient (using the Goal Attainment Scale) [69] and treatment plans (updated daily) and a discharge checklist. Post-acute follow-up to provide ongoing education and modification of the function-focused goals and care plan continues through weekly telephone contact starting within 48 h of discharge, and continuing for a total of 8 weeks, then monthly for 4 months. In Component IV, the Fam-FFC nurse works with the champions to mentor nursing staff (RN, LPN, nursing assistants) to provide Fam-FFC by role-modeling Fam-FFC, co-assessing the patient’s capability [70], reinforcing performance of and benefits of Fam-FFC at staff meetings and huddles, brainstorming about ways to overcome challenges, representing study activity to management, and observing for follow-through of the care plan [71]. The four components of Fam-FFC are described in more detail in Table 1.

**Table 1:** Description of the Family centered Function-focused Care (Fam-FFC) intervention

Component	When delivered	By whom	Description
I*.* Environmental and Policy Assessment	Beginning of the study (during the first month of study); at completion of the implementation	Fam-FFC research nurse with unit champions; recommendations for change discussed with administration	Possible modifications include development of policies for: labeling glasses/hearing aids, uninterrupted quiet times, FCG involvement in rounds, and bedside white boards to promote FCG/patient communication with the interdisciplinary team; and access to hearing amplifiers, magnifiers, activity cart/ supplies; mobility devices; noise trackers; snacks and fluids
II. Staff Education and Training (delivery options include: instructor-led PowerPoint presentations, web-based training, and one on-one review)	Beginning of the study (during the first 2 months of study)	Fam-FFC research nurse on intervention units; alternate nurse on control units	Content includes: • dementia, delirium, functional decline etiology, cognitive and functional assessment, patient communication, and evidence-based approaches to prevent cascade iatrogenesis • hospital experience and responses of the patient/family • function-focused care (rationale, incorporating FFC into routine care, specific techniques/ equipment, safety considerations, goal setting/ discharge planning) • partnerships with families (assessment of preferences, active listening, information-sharing, care planning, promoting advocacy and patient/ family engagement in decision-making, discharge planning)
III. FamPath Care Pathway	During the 12 months of implementation	Fam-FFC research nurse	Components of FamPath include: • information on the admitting condition, diagnostics, and treatment • family/patient education: provided in lay terms (cueing and motivating techniques, support of physical activity, meals, cognitive stimulation, behavioral support, and safety) linked to joint FCG/ nurse assessment (baseline cognition, physical function and social profile) • jointly developed bedside goals [69] and treatment plans (updated daily) and discharge checklist • coaching of primary nurse to communicate and provide a copy of the FamPath plan to post-acute providers • post-acute follow-up to provide ongoing education and modification of the function-focused care plan (within 48 h of discharge, weekly telephone calls for a total of 7 additional weeks, then monthly for 4 months)
IV. Ongoing Training and Motivation of Nursing Staff	Following initial education of the staff; during 12 months of implementation	Fam-FFC research nurse mentors the unit champions and nursing staff	Components include: • assistance to champions and nurses is provided on consented patients to: (a) complete the physical capability assessments [70], (b) establish and update FFC goals with input from FCGs/patients (Goal Attainment Scale) [69] and (c) develop a care plan with FCG/ patient addressing factors that impede FFC (e.g., acute illness, sedation, pain, fear/anxiety, pain, apathy, NPS, depression) • support of the unit champions to mentor nursing staff (RN, LPN, nursing assistants) includes: (a) role-modeling; (b) highlighting staff role models and positive opinion leaders; (c) garnering support by sharing success stories with nursing council and administration; (d) maintaining Fam-FFC bulletin board with updates /educational reinforcement; and (e) observing nursing staff during care interactions using the Function Focused Care Behavior Checklist [71], providing feedback to staff

Legend: *Fam-FCC* Family centered Function-focused Care, *FCG* family caregiver, *LPN* licensed practical nurse, *NPS* neuropsychiatric symptoms, *RN* research nurse

## Description of the control condition

The Attention Control condition (Fam- FFC Ed-only) consists of education of the nursing staff per manualized protocol in participating hospital units. The education is offered exactly as in treatment sites, provided by research staff not familiar with the intervention. Education of FCGs includes orientation to the hospital and traditional discharge teaching (medications/ treatments, medical follow-up).

## Treatment fidelity

Treatment fidelity is evaluated with regard to delivery of the intervention, receipt, and enactment [72, 73] as shown in Table 2. Evidence of delivery is based on completion of the *Assessment of the Hospital Environment and Policy* at the initiation of the study on hospital units and at the end of the study period. The assessment is conducted by the Fam-FFC research nurse with champions as part of the intervention [54, 55]. Likewise, completion of the *The Goal Attainment Scale* [69] is used as evidence of delivery of the intervention. Evidence of receipt is based on scoring of the scales and evidence that there is improvement in the environments/ policies at the end of the study period and that the patient’s goals have been achieved at the time of discharge and during the home follow-up period. The scoring of the goal form is completed by the Fam-FFC research nurse with input from FCGs, champions, nursing staff, and other health care professionals who work with the patient. Receipt of the intervention is based on scores on the *Knowledge of Fam-FFC test which* is a valid and reliable, 15- item, paper-and-pencil multiple choice exam that tests staff knowledge about Fam-FFC [54, 55]. A score of 80% or greater among the nurses exposed to education is evidence of receipt of the intervention for staff in both the control and intervention groups. Enactment of the intervention is based on the *FamPath Audit,* which tracks delivery of FamPath and an observation of the nurse-patient interaction daily during the course of the hospital stay, using the *Function Focused Care Behavior Checklist (FFC-BC).* The FFC-BC is a valid and reliable objective measure that includes a 19-item checklist reflecting nursing staff performance of FFC [71]. Bi-weekly meetings of the Fam-FFC research nurse with the principal investigator (PI) assure fidelity to the intervention.

**Table 2:** Treatment fidelity (italicized items: both treatment and control sites; items not bolded: treatment site only)

Focus	Data	Evidence of treatment fidelity
Delivery	Assessment of the hospital environment and policy [54, 55] (Component I)	Completion of assessments by Fam-FFC research nurse.
	*Education: percentage of nurses exposed* (Component II) [54, 55]	*80% of all nursing staff working on participating nursing units (# exposed/total # nursing staff)*
	Goal attainment forms [69] (Component III)	Forms completed on all recruited patients in treatment units
	Fam-Path Audit [54, 55] (Component III)	Completion of bedside goals and treatment plans, discharge checklist, post-acute follow-up and plan update
Receipt	*Knowledge of Fam-FFC Test* [54, 55]	*Mean score of > 80% among nursing staff after education*
	Assessment of the hospital environment and policy [54, 55]	Conducted at the beginning and end of the study at each site to evaluate for evidence of change(s) made over the course of the study
	Goal Attainment Scale [69]	Goal attainment scores incorporated into care plans
Enactment	Function Focused Care Behavior Checklist (FFC-BC) [54, 55, 71] (Component IV)	Performance of Fam-FFC by nurses based on observations of care interactions in the hospital; evaluated on at least 50% of the patients per site

## Study discontinuation

Reasons for which participants may wish to discontinue the program may be varied, e.g., relocation, lack of time, lost interest in the program, or feeling too unwell. Participants who wish to discontinue will be invited to voluntarily inform the study team by email or telephone and provide reasons for discontinuation.

## Measurement/instruments

**Fig. 2:**
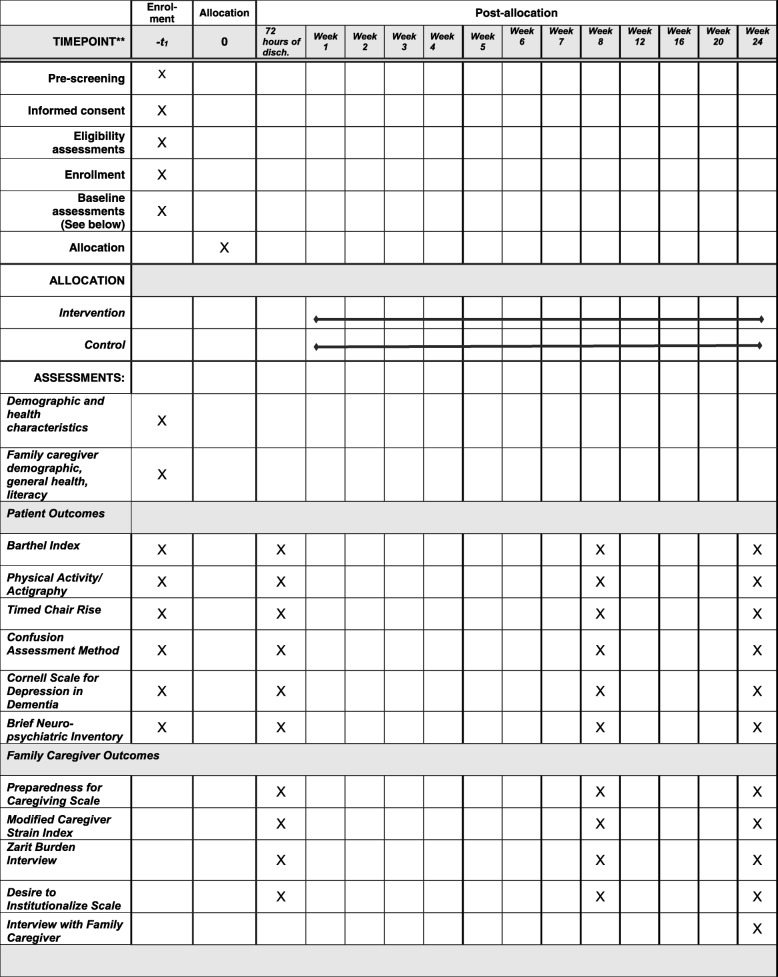
Overall schedule and time commitment for trial participants

Figure 2 illustrates the overall schedule and time commitment for trial participants in both groups. Standardized data are collected via tablet computers and uploaded to the REDCap (Research Electronic Data Capture) data entry and management system. The training of evaluators includes interrater reliability (minimum of 90%). In addition to initial reliability testing, we conduct reliability rechecks at 6 and 9 months at each intervention site. The project manager audits data on a daily basis for completeness and appropriate ranges. All data are coded with an arbitrary subject number to allow for linking of data over time. The linkage to individual names is stored in a HIPAA-protected, password-accessed, Cloud content management and file sharing system. This information will be deleted when all study-related activities are completed.

### Descriptive measures

Patient descriptive measures include age, race/ethnicity, gender, marital status, education, medical diagnoses, medications, the use of sensory and mobility devices, length of stay, and disease burden. Co-morbid conditions are classified with the *Charlson Co-morbidity Index*, a weighted index that takes into account both the number and seriousness of different co-morbid diseases, considered a valid and reliable measure of disease burden [74]. Pre-admission function (ADL performance 2 weeks prior to admission) using the *Barthel Index* [75] (also an outcome measure) is evaluated based on report from the FCG. Upon discharge, details of length of stay, use of tethers (intravenous (IV) lines, pulse oximetry, urinary catheters, etc.), physical restraint use, and discharge disposition (home or other, e.g., assisted living or skilled nursing facility) are acquired.

Cognitive domains affected by both dementia and delirium are evaluated including executive function, orientation, memory, abstract thinking, and attention, using the subdomains of the *MoCA* [59]. The use of rehabilitation therapies (number of physical therapy, occupational therapy and speech therapy sessions) and psychotropic medication prescribed during the hospitalization (drug, dose, frequency of antipsychotics, antidepressants, anxiolytics, sedatives/ hypnotics, mood stabilizers) are obtained through chart abstraction at discharge and FCG report after discharge.

FCG descriptive measures include age, race/ethnicity, gender, marital status, education, employment status, health literacy and relation to patient, acquired at the time of the first visit. General health and change in health compared to 1 year ago are assessed using two widely used items from the SF-36 [76]. Health literacy (reading) is measured with the *REALM-short form.* The REALM-short form is highly correlated with three other tests with a reported test-retest of 0.99 [76, 77]. Anxiety is evaluated with the seven-item *Hospital Anxiety and Depression Scale (HADS*) subscale for Anxiety (HADS-A) and depression with the seven-item Hospital Anxiety and Depression Scale (HADS) subscale for Depression (HADS-D) [78, 79]. Scores can range from 0 to 21 with scores categorized as follows: normal (0–7), mild (8–10), moderate (11–14), severe (15–21) for each of the valid and reliable HADS subscales. Suicidal ideation is assessed using the Ask Suicide-Screening Questions [80].

### Measures of primary outcomes

Our primary patient outcomes are physical function (activities of daily living, functional performance/ chair rise, physical activity), delirium (occurrence and severity), mood and behavior. These measures are collected within 48 h of admission to the unit (T0), within 72 h post discharge (T1), 8 weeks (T2), and 24 weeks post discharge (T3), by research evaluators via observation, input from staff, chart abstracting or interview of the patient/ FCG.

*ADLs* (activities of daily living) are assessed using the *Barthel Index* verbal report obtained from the nurse who is assigned to the patient’s care on the day of data collection. This 14-item measure of physical function assesses ability for self-care [75]. There is sufficient evidence for the reliability and validity of the Barthel Index when used with older adults [75, 81], individuals with progressive neurological conditions [82], and when proxy respondents were utilized to report the functional abilities of dementia patients [83].

*Functional performance* is evaluated with the *Timed Chair Rise,* an item of the Balance subscale of the Tinetti Gait and Balance Scale [84]. Previous work has identified this measure as a reliable and valid indication of function and strength [81]. *Physical activity* is measured for 24 h using the *MotionWatch.* The MotionWatch offers wrist-worn actigraphy that records activity in set epochs of time [85] with established reliability and validity [85–89], and is well tolerated by cognitively impaired older adults [89].

*Delirium occurrence* is evaluated using structured interview consisting of questions from the *MoCA* observation [59] (items on orientation, memory, and language are used in the assessment of delirium) and the *Confusion Assessment Method* (CAM) [90]. *Delirium severity* is assessed through an additive score for six items of the *CAM* (inattention, disorganized thinking, disorientation, memory impairment, perceptual disturbances, and psychomotor agitation/ retardation), scored as absent (0 points), present in mild form (1 point), or present in severe form (2 points). The seventh item, altered level of consciousness, is scored as alert (0 points), vigilant or lethargic (1 point), and stupor or coma (2 points). Scores range from 0 and 14, with a higher score indicating greater severity of delirium [91].

*Mood* is assessed using the *Cornell Scale for Depression in Dementia* (CSDD), a 19-item survey designed to assess depressive symptoms in individuals with dementia [92, 93]. There is sufficient evidence of reliability and validity [93]. *Behavior* is evaluated with the *Brief Neuropsychiatric Inventory* (NPI-Q), a 12-item reliable, validated informant-based assessment of neuropsychiatric symptoms and associated caregiver distress [94].

Our primary family caregiver outcomes are collected within 72 h of discharge (T1), 8 weeks (T2) and 24 weeks post discharge (T3), and include preparedness for caregiving, strain, burden, and desire to institutionalize scales: the *Preparedness for Caregiving Scale* is an eight-item instrument that asks caregivers how well prepared they believe they are for multiple domains of caregiving: physical care and emotional support, setting up support services, dealing with the stress of caregiving. Items are rated 0 (not at all prepared) to 4 (very well prepared); shows very good reliability and internal consistency [95], including in FCGs of hospitalized persons with dementia [54]. The *Modified Caregiver Strain Index (CSI)* is a 13-question tool that measures strain (financial, physical, social, psychological, and personal) related to care provision, with excellent internal consistency and reliability [96, 97]. Caregiver burden is measured with the *Zarit Burden Interview (ZBI-12)* short version, a 12-item tool that measures caregiver perception of burden has shown high internal consistency and discriminative ability in dementia caregivers [98]. The Spanish version has demonstrated good psychometric properties [99]. The *Desire to Institutionalize Scale* is a six-item scale with moderate reliability (Cronbach’s alpha = 0.694) [100].

### Qualitative data: staff nurse views of the intervention

A semi-structured interview guide is used in focus groups that explore staff nurses’ views of the effectiveness of the intervention, as well as the barriers to, facilitators of, and sustainability of the intervention. The focus groups, conducted at the conclusion of the intervention at each site, are audiotaped, transcribed, and audited for consistency with written recordings.

### Cost outcomes

We have developed cost-related data collection forms, specifically Activity Diaries, to help facilitate data collection for the activities and time to complete the activities that are above and beyond routine care for the champions, research nurse facilitators, and staff. These include staff and research nurse time and hours worked, training time including for replacement personnel), supplies, and incentives. Post-acute health care utilization by the patient is evaluated with a cumulative count of emergency room visits, number/days of hospitalizations, and long-term nursing home admissions 24 weeks after discharge, obtained from questioning of the family at each point of data collection. Health care unit charges and reimbursement rates are obtained from yearly publications from the American Hospital Associations, other health data organizations, and the published literature.

## Sample size calculations

We powered our study based on our primary endpoint, ADLs. In our pilot study, the effect size on the change score of this endpoint from baseline to 12 months is an expected Cohen’s *d* = 0.55 [54, 55]. For any cluster randomized trials, a key issue for sample size and power is the intracluster correlation coefficient (ICC) which measures the similarity of the outcomes from individuals within the same cluster. Our pilot study data showed that the ICC for ADL was small: ICC = 0.073. Our pilot work demonstrated low attrition rates (3 to 5%) and attrition may increase given that the current trial will be larger in size. We target a total sample size of 438 with 73 per inpatient unit, sufficient to detect an intervention effect comparable to the observed effect size in our pilot study, with 80% statistical power assuming a 20% attrition rate. We incorporate the following recruitment approaches to reach target sample size: (1) twice a day contact with unit staff to capture potential participants, and (2) the coordination of recruitment activity so as not to conflict with routine patient and staff activity (e.g., rounds, meals, peak personal care times). Sample retention is always a potential limitation in longitudinal studies. We address this issue by offering modest honorariums a (a US$20 gift card to FCGs at discharge from the hospital, month 2, and month 6), scheduling interviews at a time/ location convenient to the patient and FCG and rescheduling canceled interviews. We also utilize scheduling reminders and plan the data collection in such a way as to promote continuity between acute and post-acute staff.

## Analysis of aims

The intent-to-treat principle is followed to analyze data [101]. If the participant withdraws from this study, already-collected data are not removed from the study database. Initial analysis of the patient and their caregiver’s characteristics by experimental group at baseline and each follow-up time point is conducted to assess for any potential bias created by randomization and differential attrition. We perform the same analysis to compare other potential confounders on hospitalization and long-term institutionalization and identify any that are unbalanced between the intervention and the control groups. All unbalanced covariates are included in the outcome analyses to adjust for confounding. In case there are large number of unbalanced covariates, propensity score methods are used for controlling their confounding effects [102]. Other variables that are included are patient and FCG gender, race/ethnicity, age, patient co-morbidity, dementia severity, and the amount/nature of contact with the patient/ FCG (extracted from the FamPath Activity Log) as these variables are likely to impact outcomes. We anticipate that the majority (> 90%) of patients will be discharged to home (as opposed to assisted living or subacute rehab) [54, 55]; however, we plan for potential discharge to non-home settings and include discharge disposition as a covariate.

### Analyses of primary outcomes

For each patient and FCG outcome, an appropriate mixed-effects model (either a linear mixed effect (LME) or a generalized linear mixed model (GLMM)) will be identified for model-fitting depending on the distributions of the outcome variables. For delirium occurrence (a binary variable) we will use GLMMs with a logistic link function. The primary independent variables will be GROUP (Fam FCC v. control), TIME (day of admission, day of discharge, 8 weeks post discharge and 24 weeks post discharge), and SITE (for the three hospitals). The TIME × GROUP interaction will be tested for differential rate of change in the outcome. The GROUP × SITE interaction will be tested for heterogeneous hospital effects. Three-level models (level 1: time, level 2: patient, level 3 hospital) using an unstructured correlation will be fitted for the repeated measures within individuals who are nested within the same inpatient unit [103–106]. Time will be modeled continuously using day since admission so that trajectories can vary across individuals depending on time spent in the inpatient unit. This also allows us to examine non-linear time functions (e.g., quadratic) that allow the rate of change to vary over the different intervals. Covariates identified from the baseline analyses will be included in the mixed model to adjust for their influences. We will include time from admission to discharge (as a difference) at the individual level to evaluate the potential influence of hospital length of stay. We will also consider interactive effects among demographic variables of interest (e.g., age, gender of patient and FCG, ethnicity) and treatment condition to identify moderators of the treatment effects.

Bootstrap methods will be applied to the mixed-effects models in addition to the Wald test and the likelihood ratio test within the mixed-effects models [107–109]. To address the multiple endpoints issue in this trial, *p* < 0.05 for the primary hypothesis and then multiple testing procedures such as closed testing implemented in PROC MULTTEST will be applied to adjust *p* values. For missing data**,** mixed-effects models tolerate missing-at-random data using restricted estimation maximum likelihood that allows unbalanced data (e.g., missing follow-up assessments for some participants). For non-ignorable missing data, pattern mixture random-effects models can be applied to estimate the average treatment effect. Sensitivity analyses will determine if different sets of assumptions on the missing data structure lead to similar conclusions [110].

### Cost analyses

Standard micro-costing methods will be used to *estimate the cost of Fam-FCC.* Using the guidelines offered by Chatterji and colleagues [111], total intervention costs will be based on expenditures/ outlays for each of the following: (1) intervention (activity logs reporting time and cost of Fam-FFC nurse and nurse champions); (2) personnel costs (including staff training), (3) incentives for study participants; and (4) supplies/equipment costs. We will identify all of the “inputs” that go into the implementation and then assign unit costs to these inputs (wages/salaries acquired from the unit managers, cost of materials, etc.). Then, we will multiply the units of inputs by their unit costs and sum this across all of inputs to determine the total cost of implementation per setting. An average across all settings will then be calculated.

To *estimate the cost of health care for each group,* we will collect unit costs from existing literature including data published yearly by the American Medical Association, the American Hospital Association, the National Hospital Discharge Survey, and the American Managed Behavioral Healthcare Association. Similarly, these unit cost estimates for each type of health care utilization (emergency room, hospital and long-term nursing home admissions) will then be multiplied by the number of units used by each individual in the sample. A variable will be created for total health care cost for the admission and all health care services in the 24 weeks after discharge by summing across conditions while exercising care to avoid any double counting of services*. Health care and total cost savings* will be calculated for each treatment condition. Mean costs and savings will be computed and subsequently tested for statistical significances across conditions using a *t* test. In calculating cost, we will also take into account fixed versus variable costs adjusting for variation over time in wages, benefits, and supplies using a standard micro-costing approach. We will then use multivariable regressions to determine whether Fam-FCC is significantly related to lower costs of health care and total cost savings controlling for covariates. Moreover, we will compare health care costs in terms of changes over time with paired a *t* test (within-group differences) and with parametric analysis of variance (between group differences).

### Assessment of the cultural appropriateness of Fam-FCC

We employ the Ecological Model (EM) originally developed by Bernal and colleagues as a framework to assess the cultural appropriateness of the Fam-CC intervention [112–114], and to refine Fam-FCC for future studies. The EM posits that there are eight dimensions to consider when assessing the cultural appropriateness of an intervention. As applied to Fam-FCC, we consider the following: (1) *language:* did the family receive the intervention information in terminology, vernacular in which they felt comfortable? (2) *persons*: in how many of the 438 dyads did the nurse champion reflect ethnic group of the family? (3) *metaphors*: use of cultural terms equivalent to those used by participants, (4) *content*: were the values, customs, and traditions shared by the ethnic or minority group apparent in the intervention? (5) *concepts*: were caregiving concepts congruent with cultural norms? (6) *goals*: were intervention goals congruent with family cultural norms and goals? (7) *methods*: delivery of the intervention is culturally appropriate, and (8) *context*: does the intervention consider the family’s socio-community context? We will implement this EM assessment with four components, described in Table 3.

**Table 3:** Components of the cultural appropriateness assessment of Fam-FFC [112–114]

EM Model Component	Sample/Data Source	Analysis
I. Family caregivers’ (FCG) experiences of Ecological Model (EM) model constructs (categories 1,3,4,5,6,8)	FCGs who self-identify as black, Latino, Asian, and white will be randomly selected (approximately 10% of families from each ethnic group; if theoretical saturation is not reached, interviews will continue until saturation is reached.) A draft semi-structured interview guide will be refined with the input of a hospital patient / family council	Qualitative content analysis of audiotaped, transcribed interviews conducted at the 6-month post-hospital discharge home visit. Trustworthiness [113] is enhanced by the team’s methodological expertise, random sampling, rigorous analytic approaches, member checking, and a detailed audit trail
II. Nurse champion experiences of EM model constructs (categories 1,3,4,5,6,8)	The 6 champions (2 per setting) will be interviewed using a semi-structured interview guide	Qualitative content analysis of audiotaped, transcribed interviews at conclusion of intervention at each site
III. Evaluation of measures for reliability and validity for the ethnic groups represented in the study (category 7)	1) The internal consistency of the caregiver outcome measures as well as relationships with known correlates (e.g., educational level) will be evaluated in the first 20 Spanish-speaking respondents/ 2) Both content and face validity of each measure will be discussed in participant interviews outlined in Component I above	Cronbach’s alphas for caregiver outcomes will be assessed. The participants will be asked to assess their perceptions of measures and identify potential cultural gaps in measurement content in Component I above
IV. Assessment of whether ethnic concordance moderates the relationship between treatment group and patient/ family outcomes (category 2)	The ethnicities of nurse champions and each family will be documented as concordant or not	Concordance will potentially be used as a covariate in analyses for specific aims 1, 2

We will analyze the data of the focus groups with nurses that explores barriers, facilitators, and sustainability of Fam-FFC through qualitative content analysis of audiotaped, transcribed interviews conducted at the conclusion of the intervention at each site. Three members of the research team will code the data and compare results until consensus is reached. Codes will then be reduced to themes. Trustworthiness will be supported by the team’s methodological expertise, rigorous analytic approaches, member checking, and a detailed audit trail [113].

## Monitoring the study

The Data Safety Monitoring Committee (DSMC) is chaired by a researcher who has extensive expertise in data safety and monitoring, clinical trials, and dementia caregiving research including bioethical considerations. The additional members of the DMSC include two biostatisticians who have experience on a DSMC and a nurse clinician with extensive experience in behavior-change research and care of older adults with dementia. The DSMC will meet at least annually via web conferencing; all members of the investigative team are invited to participate in open meetings. If necessary, the DSMC chair may hold a closed meeting, to be attended only by the committee and representative from the NIA. The first meeting of the DSMC held prior to recruitment of participants focused on review of the study protocol and recruitment plan, consideration of participant burden, and a review of our plan for participant safety and comfort. The subsequent meetings focus on the progress of the study with regard to data quality and timeliness, participant consent, accrual and retention of participants, participant risk versus benefit, performance of intervention units, and any adverse events that occur during the course of the study to date. One or more major adverse events will also trigger a DSMC meeting. At the end of each meeting, the committee makes recommendations to the NIH, Institutional Review Board(s), and the PI and investigative team concerning continuation or conclusion of the trial.

## Discussion

The care and services provided in the hospital have a profound and permanent effect on persons with ADRD and their families, in terms of not only their inpatient experience, but also their ongoing functioning, relationships, wellbeing, quality of life and the fundamental decisions that are made about their future. The negative consequences of an acute illness or injury often begin prior to the hospitalization [115] and persist well into the post-acute period, resulting in increased risk for care dependency, FCG burden, long-term nursing home stay, resource consumption, and cost. The proposed study builds upon pilot work [54, 55] in the acute care setting and focuses on improving functional and behavioral outcomes that impact the quality of life for both FCGs and patients with dementia, a population that is often excluded from acute care research.

This study is limited in that it will be conducted in only three acute care facilities in a single region. There is also the risk of carryover between treatment and control units given that the staff nurses from different units may talk to each other during non-work time in a social setting (such as in the cafeteria, parking lot, etc.). Measurement is limited as some measures are dependent on input from staff that may not know the patient that well (e.g., the Barthel Index). Despite challenges, this study constitutes an important step in quantifying the long-term, positive impact of Fam-FFC among patients with very mild to moderate cognitive impairment, as well as their FCGs. If the full Fam-FFC demonstrates positive results, we will need to evaluate the efficacy of different components and dosing of the intervention in future research.

The strengths of the study include the use of facility-based champions and a rigorous treatment fidelity plan. The Fam-FFC intervention has the potential to reduce poor health outcomes that are major sources of today’s spiraling health care costs. This study should have direct implications for clinical practice and informing policy related to effective care delivery for persons with dementia in acute care, a neglected area of research and program development. The societal implications of helping older individuals with ADRD avoid functional decline, adverse events, long-term nursing home admissions, and hospitalizations are enormous in terms of aging in place, quality of life, cost, and caregiver burden. The study findings will be relevant for other areas of behavior-change research in acute care, specifically those related to engaging patients and families in health care planning, delivery, and evaluation (Additional file 1).

## Trial status

Recruitment began in November 2017 and is expected to finish in October 2021, approximately.

## Additional file

Additional file 1: Standard Protocol Items: Recommendations for Interventional Trials (SPIRIT) 2013 Checklist. (PDF 175 kb)

## Abbreviations

ADLs: Activities of daily living
ADRD: Alzheimer’s disease or a related dementia
CAM: Confusion Assessment Method
CDR: Clinical Dementia Rating Scale
CSDD: Cornell Scale for Depression in Dementia
CSI: Modified Caregiver Strain Index
DSMC: Data Safety Monitoring Committee
EM: Ecological Model
ESC: Evaluation to Sign Consent
Fam-FFC Ed-only: Fam- FFC Education-only
Fam-FFC Nurse: Family centered Function Focused Care nurse
Fam-FFC: Family centered Function-focused Care
FAQ: Functional Activities Questionnaire
FCG: Family caregiver
FFC-BC: Function Focused Care Behavior Checklist
HADS: Hospital Anxiety and Depression Scale
HADS-A: Hospital Anxiety and Depression Scale subscale for Anxiety
HADS-D: Hospital Anxiety and Depression Scale subscale for Depression
ICC: Intracluster correlation coefficient
LAR: Legally authorized representative
MoCA: Montreal Cognitive Assessment
NIA: National Institute of Aging
REDCap: Research Electronic Data Capture
SCT: Social Cognitive Theory
